# Supplementary material for: Identification and functional analysis of non-coding regulatory small RNA FenSr3 in Bacillus amyloliquefaciens LPB-18
Source: PeerJ. 2023 May 15;11:e15236. doi: 10.7717/peerj.15236 (PMC10194069; doi:10.7717/peerj.15236)
Supplement: Supplemental Information 4 [file peerj-11-15236-s004.zip › KO/CK-vs-T1_map/map04150.html]

KEGG PATHWAY: mTOR signaling pathway - Reference pathway


|  |  |
| --- | --- |
| **mTOR signaling pathway - Reference pathway** |  |

[
Pathway menu
| Organism menu
| Pathway entry
| Show description
| User data mapping
]

The mammalian (mechanistic) target of rapamycin (mTOR) is a highly conserved serine/threonine protein kinase, which exists in two complexes termed mTOR complex 1 (mTORC1) and 2 (mTORC2). mTORC1 contains mTOR, Raptor, PRAS40, Deptor, mLST8, Tel2 and Tti1. mTORC1 is activated by the presence of growth factors, amino acids, energy status, stress and oxygen levels to regulate several biological processes, including lipid metabolism, autophagy, protein synthesis and ribosome biogenesis. On the other hand, mTORC2, which consists of mTOR, mSin1, Rictor, Protor, Deptor, mLST8, Tel2 and Tti1, responds to growth factors and controls cytoskeletal organization, metabolism and survival.

|  |  |  |
| --- | --- | --- |
| Reference pathway | 184% 150% 122% 100% 82% 67% 55% | 图片下载 |
